# Gender-based violence and its associated factors among internally displaced women in Northwest Ethiopia: a cross-sectional study

**DOI:** 10.1186/s12905-023-02306-2

**Published:** 2023-04-06

**Authors:** Keralem Workie, Techilo Tinsae, Endalamaw Salelew, Biksegn Asrat

**Affiliations:** 1 Department of Psychiatry, College of Medicine and Health Sciences, Hawassa University, Hawassa, Ethiopia; 2 Department of Psychiatry, College of Medicine and Health Sciences, University of Gondar, P.o.Box: 196, Gondar, Ethiopia

**Keywords:** Conflict, Gender-based violence, Internally displaced women, Northwest Ethiopia

## Abstract

**Background:**

Gender-based violence (GBV) is a common human right violence in conflict-affected communities. Women with GBV are prone to experience mental health problems such as post-traumatic stress disorder, depression, and anxiety. However, there is a paucity of evidence as to what extent the problem is affecting internally displaced women (IDW) in Ethiopia. This study aimed to assess the prevalence of GBV and its associated factors among IDW in Northwest Ethiopia.

**Methods:**

A cross-sectional study was conducted among IDW residing at three humanitarian sites from May to June 2022 in Northwest Ethiopia. Study participants were selected using a stratified simple random sampling technique from the three sites. GBV was assessed using a 6-item Assessment Screen to Identify Survivors Toolkit questionnaire for Gender-based violence (ASIST-GBV). Data were analyzed using binary logistic regression. All variables with a *p*-value of ≤ 0.05 in the multivariable analysis were defined to have a statistically significant association with GBV at a 95% confidence interval (CI).

**Results:**

Of 424 approached candidates, 412 (97.2%) of them participated in the study. A one-year prevalence of GBV was 37.9% (95%CI = 33.2–42.6) among IDW in Northwest Ethiopia. The mean age of the participants was 31.3 (± 7.6) years. Young women, 18–24 years old (AOR = 3.52, 95%CI = 2.15–5.34, *p* ≤ 0.001) and 25–29 years old (AOR = 2.41, 95%CI = 1.57–3.24, *p* ≤ 0.001) had a statistically significant association with GBV. Moreover, having no social protection (AOR = 3.18, 95%CI = 2.65–6.22, *p* ≤ 0.001), being current alcohol user (AOR = 2.54, 95%CI = 1.22–4.78, *p* ≤ 0.001) and being single in marital status (AOR = 1.69, 95%CI = 1.18–2.87, *p* < 0.01) showed a statistical association with GBV.

**Conclusion:**

We found a high prevalence of GBV among IDW in Northwest Ethiopia which indicates that IDW are prone to GBV. We call for immediate action and special attention to young women in conflict-affected parts of Ethiopia. It is crucial to establish a system that ensures the safety, security, and well-being of women in humanitarian settings.

**Supplementary Information:**

The online version contains supplementary material available at 10.1186/s12905-023-02306-2.

## Introduction

Ethiopia has experienced numerous social, political, and economic crises due to internal conflicts [1, 2]. There has been a large-scale internal displacement following internal conflicts in Ethiopia, particularly since 2018. As the 2019 Internal Displacement Monitoring Centre (IDMC) report, more than 2.9 million people were internally displaced in Ethiopia and this record was the highest in the world [3]. An emergency site assessment by International Organization for Migrants (IOM) showed that as of September 2021 there were 2.6 million internally displaced people in the three regions (Tigray, Amhara, and Afar) in North Ethiopia [3]. Humanitarian reports indicate that around 80% of internally displaced people are women and children who are at risk for gender-based violence (GBV) [4, 5].

GBV is a human right violence by a perpetrator against a person because of his/her gender or sex that results in physical, sexual, or psychological harm or suffering to a person [6–8]. GBV can be committed by intimate partners, family members, or a group of strange people [9]. Young women and girls are particularly prone to GBV in humanitarian settings [10, 11]. GBV remains the most concerning issue globally particularly in conflict affected parts of the world. Over 60% of women and girls experience GBV in humanitarian settings in low- and middle-income countries (LMICs).

Several factors could increase the risk for GBV such as i) personal factors including young age, illiteracy, and not being married [12]; ii) socio-cultural factors such as poverty, unemployment, rural residence, no social support/protection, having a risky occupation and patriarchal culture [13]; iii) behavioral factors such as alcohol and other substances use [14–17]. Young women and girls are particularly vulnerable to GBV in conflict-affected communities.

The consequences of GBV are devastating that could have life-long traumatic sequala on a person’s life. Women who experienced physical trauma could acquire sexually transmitted diseases and unwanted pregnancies. GBV could also have psychological trauma that exposes the affected person to mental health problems such as anxiety, depression, and post-traumatic stress disorder (PTSD) [18–21]. Moreover, GBV could affect the social well-being which could lead to loneliness, social withdrawal, and a victim mentality in the affected person.

Despite enormous impacts, GBV is overlooked and under-investigated in humanitarian settings in Ethiopia. There is a need for data on to what extent women are experiencing GBV and what contributes to the incidence of the problem in conflict-affected communities in Northwest Ethiopia. The aim of this study was to assess the magnitude of GBV and its associated factors among IDW in Northwest Ethiopia.

## Method

### Study design and sites

A humanitarian setting-based cross-sectional study was conducted from May to June 2022 among IDW in three sheltering sites (in Gondar, Dabat, and Debark towns) in Northwest Ethiopia.

Site 1 is located in Gondar town and there were about 2,857 people sheltering (1,552 of them were female) in this site. Site 2 is located in Dabat town, 53 km away from Gondar town to the North. There were about 2,084 people (1,048 of them are female) in this camp. Site 3 is located in Debark town, 90 km away from Gondar town to the North. There were about 2,423 people (2,105 of them were female) living in this site. In this study, we included all women with age 18 years and above. Women who were in serious health conditions such as physical illness and with verbal communication problems were excluded.

### Sampling procedure and recruitment 

The sample size was calculated using a single population proportion formula taking 50.6% prevalence of GBV in Bokolomayo refugee site in eastern Ethiopia [22], considering a 95% confidence level and 5% margin of error.

The study participants were recruited using a stratified simple random sampling technique with a computer-generated method. Proportional allocation was used to take a representative sample from the three sites. Study participants were selected using the computer-generated random numbers method. Every internally displaced person had identification information that consists of identification number and living room number. The lists of participants with their identification numbers were collected from the camp administrators, then the identification numbers were entered into STATA 16 to select study participants. Information on where candidate participants are living was captured. Then each candidate participant was approached by data collectors in their living room. Finally, those who are willing to participate in the study were signed written informed consent and then interviewed.

### Measurement

Structured questions were used to collect socio-demographic information such as age, marital status, education level, and so on. Information related to GBV was collected using a 6-item Assessment Screen to Identify Survivors Toolkit questionnaire for Gender Based Violence (ASIST-GBV) which was validated in Bokolomayo refugee site in eastern Ethiopia [22]. The tool includes questions related to emotional violence, physical violence, forced sex, coercive sex for survival, forced pregnancy, and forced marriage. ASIST-GBV has strong psychometric properties and good reliability that had Cronbach’s α = 0.77. The tool has been used commonly to assess female GBV in humanitarian settings in LMICs. GBV was defined as having at least one ‘yes’ response from the 6 items of ASIST-GBV [22].

Behavioral factors were assessed using a semi-structured WHO ASSIST (Alcohol, Smoking, and Substance Involvement Screening Test) instrument [23]. Perceived social support was assessed using a 12-item multidimensional scale of perceived social support (MSPSS) which was initially designed to assess PSS from three different subscales: family scale, friends scale, and significant others scale [24]. The scores of each item were summed to calculate the total score of perceived social support (PSS) and the total scores could range from 12 to 84 points. Scores between 12–35, 36–60, and 61–84 indicate low, moderate, and high PSS respectively [24]. Respondents were asked to rate the level of social support they are receiving from option 1 “very strongly disagree” to 7 “very strongly agree” [25]. Depression was measured using the Patient Health Questionnaire (PHQ-9) assessment tool which consists of nine questions on depression symptoms over the last 2 weeks [26]. The tool includes the following response options: 0 “not at all”, 1 “several days”, 2 “more than half the days”, and 3 “nearly every day”. The sum of items could range between 0 to 27 and having a score of ≥ 10 was used to indicate at least moderate depression [26]. Anxiety was measured using the Generalized Anxiety Disorder 7 (GAD-7) instrument which consists of seven questions on anxiety symptoms over the last 2 weeks [26]. The tool has the following response options 0 “not at all”, 1 “several days”, 2 “more than half the days”, and 3 “nearly every day” and the total scores could range from 0 to 21. A score of ≥ 10 indicates at least moderate anxiety [26]. PTSD was measured using the Post-Traumatic Stress Disorder Checklist for DSM-5 (PCL-5) [27]. The PCL-5 is a standardized instrument and is a reliable measurement for PTSD which has been used in humanitarian settings in LMICs [28, 29].

### Data collection procedures 

Data were collected by trained psychiatric nurses using an interviewer-administered questionnaire Data collection was supervised by the principal investigator (KW). Data collectors approached every selected participant to explain the purpose of the study and to ask whether she is interested to participate in the study. Candidate participants who agreed to participate signed the informed consent and were taken to a private place for the interview. Data collectors interviewed each study participant using a structured questionnaire that included sociodemographic information, and questions related to GBV, depression, anxiety, PTSD, and behavioral factors.

### Data analysis

The collected data were checked, cleaned, and analyzed using STATA version 16. Descriptive statistics was used to summarize the data in the form of mean, median, and standard deviation (SD). Then the variables were recoded and computed to satisfy the binary logistic regression model. Initially, the association of each independent variable with GBV was examined using bivariable analysis. Then all candidate variables that had an association with GVB at ≤ 0.2 in bivariable analyses were taken to the multivariable logistic regression analyses. Finally, all variables with a *p*-value of ≤ 0.05 in multivariable logistic regression were defined to have a statistically significant association with GBV. The strength of association was estimated using an adjusted odds ratio (AOR) with a 95% confidence interval (CI).

## Results

### Socio-demographic characteristics

Of 424 approached candidates, 412 (97.2%) of them participated in the study. The mean age of the study participant was 31.3 (SD ± 7.6) years, and the youngest and the oldest age were 18 and 50 years old respectively. Over half (53.9%, *n* = 222) of the study subjects were married whereas 74.8% (*n* = 308) reported having no social protection at the current sheltering sites. In terms of educational status, 49.3% (*n* = 203) attended elementary school (Table 1).

**Table 1:** Sociodemographic characteristics of internally displaced women in Northwest Ethiopia, 2022 (*n* = 412)

Variables	Category	N(%)
Age	18–24	85(20.6)
	25–29	90(21.8)
	30–34	93(22.6)
	35–39	86(20.9)
	40–44	28(6.8)
	> 45	30(7.3)
Marital status	Single	60(14.6)
	Married	222(53.9)
	Divorced	90(21.8)
	Widowed	40(9.7)
Presence of children	No	91(22.1)
	Yes	321(77.9)
Social protection^a^	Yes	104(25.2)
	No	308(74.8)
Educational status	Not formal education	144(34.9)
	Elementary	203(49.3)
	Secondary and above	65(15.8)
Religion	Orthodox	383(92.9)
	Others	29(7.0)
Previous residence	Urban	365(88.6)
	Rural	47(11.4)
Previous occupation	Merchant	165(40.1)
	House wife	141(34.2)
	Student	37(8.9)
	Farmer	30(7.3)
	Daily labor	23(5.6)
	Others	16(3.9)

Other religions refer to Muslim and Protestant. Other occupations refer to public employed and home maid

^a^Social protection was defined as having a sibling, parent or any other relative who could protect them from violence

### Behavioral factors

In the current study, half of the study participants (50.2%, *n* = 207) had a history of lifetime alcohol use and 23.3% (*n* = 96) of them were current alcohol users (Table 2).

**Table 2:** Behavioral factors among internally displaced women in Northwest Ethiopia, 2022 (*n* = 412)

Variables	Categories	N(%)
Life time alcohol use	Yes	207(50.2)
	No	205(49.8)
Life time Khat use	Yes	17(4.1)
	No	395(95.9)
Current alcohol use	Yes	96(23.3)
	No	316(76.7)
Current Khat use	Yes	5(1.2)
	No	407(98.8)

### Mental health problems and psychosocial factors

Of the study participants, 53.2% (*n* = 219) of them had depression, 51.0% (*n* = 210) had anxiety and 56.1% (*n* = 231) had PTSD symptoms. One-third of study participants (64.6%, *n* = 266) reported that they received low PSS (Table 3).

**Table 3:** Mental health problems and psychosocial factors among internally displaced women in Northwest Ethiopia, 2022 (*n* = 412)

Variables	Categories	N(%)
Depression	Yes	219(53.2)
	No	193(46.8)
Anxiety	Yes	210(50.9)
	No	202(49.0)
PTSD	Yes	231(56.1)
	No	181(43.9)
Childhood abuse	Yes	28(6.8)
	No	384(93.2)
PSS	low	266(64.6)
	Moderate	126(30.6)
	High	20(4.8)

*PSS* Perceived social support, *PTSD* Post-traumatic stress disorder

### Prevalence of gender-based violence

We did Goodness-of-Fit test before conducting bivariable logistic regression analysis. We found that the Hosmer–Lemeshow chi-square was 0.75 which ensures that the model is fit for analysis. Moreover, log likelihood, log likelihood ratio and pseudo-R-square were -226.16, 0.94 and 0.17 respectively.

The overall prevalence of GBV was 37.9% (*n* = 156) with 95%CI (33.2%-42.6%). The prevalence of threat of violence, physical violence, and forced sex were 29.1% (*n* = 120), 15.8% (*n* = 65), and 6.3% (*n* = 26) respectively.

GBV was statistically associated with young women in age groups of 18–24 and 25–29, being single, absence of social protection, and current alcohol use in the multivariable logistic regression. Young women within the age range of 18–24 years old had 3.52 times higher odds GBV than those aged ≥ 40 years old (AOR = 3.52, 95%CI = 2.15–5.34, *p* ≤ 0.001). Young women within the age range of 25–29 years old had 2.41 times higher odds of GBV than women aged ≥ 40 years old (AOR = 2.41, 95%CI = 1.57–3.24, *p* ≤ 0.001). In relation to marital status, single women had 1.69 times higher odds of GBV than married women (AOR = 1.69, 95%CI = 1.18–2.87, *p* ≤ 0.01). In this study, those women who had no social protection at sheltering sites had 3.18 times higher odds of GBV than women who had social protection (AOR = 3.18, 95%CI = 2.65–6.22, *p* ≤ 0.001). From behavioral factors, women who are current alcohol users had 2.54 times higher odds of GBV than those who were not using alcohol currently (AOR = 2.54, 95%CI = 1.22–4.78, *p* ≤ 0.001) (Table 4).

**Table 4:** Bivariable and multivariable analysis: Factors associated with gender-based violence among IDW in Northwest Ethiopia (*n* = 412)

Variables	Category	GBV	COR (95%CI)	AOR (95%CI)	Variables
		Yes	No		
Age	18–24	55	50	3.81(2.34,5.58)	3.52(2.15,5.34)***
	25–29	40	50	2.77(1.68,3.43)	2.41(1.57,3.24)***
	30–34	29	64	1.57(0.96,2.01)	1.21(0.89,1.93)
	35–39	19	67	0.98(0.45,1.78)	0.63(0.12,1.13)
	≥ 40	13	45	1	
Marital status	Single	32	28	1.84 (1.04,3.27)	1.69 (1.18,2.87)**
	Married	85	137	1	
	Divorced	24	66	0.59 (0.34,1.01)	0.43 (0.27,1.32)
	Widowed	15	25	0.97 (0.48,1.94)	0.88 (0.58,3.46)
Social protection	Yes	16	88	1	
	No	140	168	4.58 (2.57,8.17)	3.18 (2.65,6.22)***
Life time alcohol use	Yes	94	113	1.92 (1.28,2.88)	1.34 (0.77,2.30)
	No	62	143	1	
Current alcohol use	Yes	57	39	3.20 (1.99,5.13)	2.54 (1.22,4.78)***
	No	99	217	1	
Childhood abuse	Yes	16	12	2.32 (1.07,5.05)	1.48(0.32,3,16)
	No	140	244	1	
Depression	Yes	103	116	2.34 (1.55,3.54)	0.69(0.35.1.49)
	No	53	140	1	
PTSD	Yes	102	129	1.85 (1.23,2.80)	0.98(0.57,1.83)
	No	54	127	1	

*Abbreviations: AOR* Adjusted odds ratio, *COR* Crude odds ratio, *CI* Confidence interval, *GBV* Gender-based violence, *PTSD* Post-traumatic stress disorder

Key: *** indicates *p*-value of ≤ 0.001, **indicates *p*-value of < 0.01

## Discussion

This study investigated the prevalence of GBV and examined its associated factors in humanitarian settings in Ethiopia. The current study found a 37.9% (95%CI = 33.2%-42.6%) prevalence of GBV in the last year among IDW in Northwest Ethiopia. In the multivariable analysis, GBV was independently associated with young age (18–24 and 25–29 years old), single marital status, having no social protection and current alcohol use.

The 37.9% prevalence of GBV in the current study was comparable with similar study findings from low-income countries such as 35.6% in Somalia [30] and 33.3% in Papua New Guinea [18]. However, it was lower than 50.6% and 63.4% prevalence of GBV in humanitarian settings in eastern Ethiopia and Colombia [22]. GBV is expected to be more common among teenagers than adults due to their vulnerability and lower self-protection capability. Hence, studies that included teenagers, for example, the study conducted in eastern Ethiopia included teenagers aged 15 years and above, could find a higher prevalence of GBV than the finding of our study. In addition, the use of different assessment tools to detect GBV could underestimate or overestimate GBV. More importantly, there could be underreporting of GBV in countries with a patriarchal culture, and similar cultures are common in Northern parts of Ethiopia. Additionally, the shorter stay in humanitarian sites and the presence of good security and legal protection in humanitarian shelters in Northwest Ethiopia could contribute to a lower prevalence of GBV in the current study compared to study findings from eastern Ethiopia and Colombia.

In the current study, young women with age between 18–24 and 25–29 years old had higher odds of GBV than women with age ≥ 40. This finding is consistent with previous study reports of an inverse association between age and GBV in which the risk of GBV is much higher in adolescents and youngsters than in older adults [31]. This result is also supported by another study finding which reported that GBV survivors are commonly at a young age [12]. This could be further explained by a culture that legitimizes GBV referring to the dominant position of men and low literacy of young women on gender equality and women right [32, 33].

GBV was also statistically associated with being single in marital status that single women had higher odds of GBV than married women. This result is in agreement with a finding of another study that reported that those who are married had a lower risk for GBV than single or unmarried women [31]. In our opinion, married women could have lower opportunity to socialize with males and could have lesser risk for GBV than single or unmarried women.

The current study found that those participants without social protection at the sheltering sites had higher odds of GBV than those who were staying with their siblings, parents, or any other relatives. Some perpetrators could afraid of committing violence against a woman who has guardians such as a father, brother, or other relatives [34], because guardians could take physical measures against perpetrators.

This study further demonstrated that current alcohol use had a statistically significant association with GBV. This finding is supported by the reports of previous studies [15–17, 35, 36]. Alcohol influences decision-making capacity and exposes women to harassment, and physical and sexual abuse. However, since the causal direction is unknown, GBV could also lead to the harmful use of alcohol and other substances. Because women affected by GBV could use alcohol to cope with psychological distress. For this reason, it is difficult to make a conclusion on which one is the cause and the effect in the current study design [36].

GBV is a key concern in conflict affected and humanitarian settings in Ethiopia. As ethnic conflict and political instability is escalating, women and girls are increasingly prone to GBV and serious health problems. Although GBV remains public concern, Ethiopia’s current gender-sensitive legal frameworks do not adequately control GBV and nor are consistent with the international policies [37, 38]. Therefore, GBV need to be incorporated in the national policies adequately to design prevention strategies and action plans to protect women and children particularly in humanitarian settings.

Our study has several strengths such as it used a standardized tool to assess GBV and used female interviewers to minimize underreporting of certain sensitive traumatic events, such as rape or sexual abuse. However, the results of this study should be interpreted with caution as a cross-sectional study could not identify the cause-and-effect relationship between GBV and independent variables.

## Conclusion 

This study found a high prevalence of GBV in the last year among IDW in Northwest Ethiopia. Young women, with single marital status, no social protection, and who use alcohol were prone to GBV. This study calls for immediate action and special attention to young women in conflict-affected parts of Ethiopia. Women affected by GBV need a timely collaborative intervention to facilitate collective healing that includes medical, psychological, and social rehabilitation. It is also crucial to establish a system that ensures the safety, security, and well-being of women in humanitarian settings. The findings of this study further warranted the need to design risk prevention programs for harmful use of alcohol in humanitarian settings. Also, more evidence is needed on magnitude and risk factors of GBV to designing interventions and supporting mechanisms for women affected by GBV.

## Supplementary Information

**Additional file 1.** Assessment Screen to Identify Survivors Toolkit questionnaire for Gender Based Violence (ASIST-GBV) Gender-Based Violence Assessment tool.

## Data Availability

The datasets used in this study are available from the corresponding author on reasonable requests.

## Abbreviations

AOR: Adjusted Odds Ratio
ASIST: Assessment Screen to Identify Survivors Toolkit
ASSIST: Alcohol, Smoking, and Substance Involvement Screening Test
DSM: Diagnostic and Statistical Manual
GAD: Generalized Anxiety Disorder
GBV: Gender Based Violence
IDMC: Internal Displacement Monitoring Centre
IDP: Internally Displaced Person
IOM: International Organization for Migrants
IPV: Intimate Partner Violence
MSPSS: Multidimensional Scale of Perceived Social Support
NPV: Non-Partner Violence
PCL: Post-traumatic stress disorder Check List
PHQ: Patient Health Questionnaire
PTSD: Post-Traumatic Stress Disorder
WHO: World Health Organization
