# Supplementary material for: Gender-based violence and its associated factors among internally displaced women in Northwest Ethiopia: a cross-sectional study
Source: BMC Womens Health. 2023 Apr 6;23:166. doi: 10.1186/s12905-023-02306-2 (PMC10080783; doi:10.1186/s12905-023-02306-2)
Supplement: Supplementary file 1 — Additional file 1. Assessment Screen to Identify Survivors Toolkit questionnaire for Gender Based Violence (ASIST-GBV) Gender-Based Violence Assessment tool. [file 12905_2023_2306_MOESM1_ESM.docx]

Assessment Screen to Identify Survivors Toolkit questionnaire for Gender Based Violence (ASIST-GBV) Gender-Based Violence Assessment tool

| SN | In the past 12 months, | Yes | No |
| --- | --- | --- | --- |
| 201 | Have you been threatened with physical or sexual violence by someone in your home or outside of your home? |  |  |
| 202 | Have you been hit, punched, kicked, slapped, choked, hurt with a weapon, or otherwise physically hurt by someone in your house or outside of your house? |  |  |
| 203 | Have you been forced to have sex against your will? |  |  |
| 204 | Were you ever forced to have sex to be able to eat, have shelter, or have sex for essential services because you or someone in your family would be in physical danger if you refused? |  |  |
| 205 | Were you ever physically forced or made to feel that you had to become pregnant against your will? |  |  |
| 205A | If yes, are you currently pregnant because of that? |  |  |
| 206 | Were you coerced or forced into marriage (or to partner with someone)? |  |  |
